# A Case of Pregnancy Attended by a Remarkable Discharge from the Uterus, Followed by a Safe Delivery

**Published:** 1841-02

**Authors:** S. E. Leonard

**Affiliations:** New Albany, Indiana


ART. II.—*A Case of Pregnancy attended by a remarkable discharge from the Uterus, followed by a safe Delivery.* By S. E. LEONARD, M. D., of New Albany, Indiana.

ABOUT the 1st of September, 1840, I was called to visit Mrs. N. in her fifth pregnancy. She supposed herself to be between the fifth and sixth months of uterogestation; but of this she was entirely uncertain, judging alone from her having quickened about the 1st of August. Her menses observed their regular periodical return until within the last six or eight weeks, as they had done during her last pregnancy, which resulted in the birth of a child at the full period. I found her discharging water from the vagina, which gushed forth copiously, unattended with pain or dilatation of the os tincæ. I also found the neck of the uterus elongated as in the sixth month. I enjoined rest in the horizontal position, and administered an opiate. The discharge ceased in a few hours, and the next day found my patient comfortable.

*September 9th.* After one week's intermission, the water again began to pour from the vagina, much more copiously than during the first attack, accompanied with a sense of uneasiness, and bearing down, rather than pain, in the pelvic region. To use her own language, she "felt as if the whole contents of her abdomen would come from her." For a few days past, she had been subject to periodical returns of fever, commencing every afternoon. Bowels torpid. Gave a mercurial cathartic, after the operation of which she had no return of febrile paroxysm. A very visible diminution of the abdominal tumour had taken place, in consequence, no doubt, of the immense discharge of water from the uterus, which in the two attacks must have been several quarts. Continued the opiates after the operation of the purgative, and still enjoined the horizontal posture. The flow of water continued, with occasional intermissions, for about forty-eight hours, at the end of which time a very considerable quantity of sanguineo-purulent matter was discharged.

*11th.* The abdominal tumour almost entirely subsided. Says she feels plainly motions of the fœtus.

*12th.* I learnt from her to-day, that she increased much more rapidly in size, than she had ever done in either of her former pregnancies. In one month from the first appearance of uterine enlargement, she was as large, or larger, than ever before at the time of her accouchement.

*28th.* Was again called in consequence of a return of the discharge of water, which had abated for about two weeks, and learnt, that for the last twelve hours water had been flowing from the vagina in almost a continuous stream. Patient informed me that since it last ceased, her size had remained almost stationary, until about three days since, when her size began to increase most rapidly, and, in her own language again, "she became a complete burden to herself." The discharge commenced with a sudden gush. She still feels plainly fœtal movements. Upon making an examination can readily trace circumscribed enlargement of the uterus. Os tincæ not at all dilated; distinctly felt the water flowing from it. No uterine pain. This last discharge produced great prostration, and nervous irritability. She had taken rhubarb and soda in the morning, which operated slightly. Prescribed opium, 1 gr. every four hours.

The discharge continued longer and more profusely, than in either of the preceding attacks, but again ceased entirely.

*October 2d.* The respite was of short duration, as she was again attacked with the flowing of water, which continued for several hours; the latter part of which time it was highly colored with blood. This, though quite profuse, had but little effect in reducing the size of the abdomen.

4*th.* The water, to-day, is of a deep yellow color, and she thinks much more copious than ever before. I am well satisfied that she passed more than a quart of water per diem, perhaps double the quantity, for four or five days successively; on the fourth it was intermixed with lumps of matter, of a caseous appearance, as large as a filbert. Urinary secretion almost entirely suspended, not voiding it more than once in twenty-four hours, and not exceeding two or three ounces at a time. General health better. Water from vagina still of a deep yellow color.

10*th.* Found my patient in great spirits, sitting in arm chair contrary to directions, and quite comfortable. The evacuation entirely ceased, for a few hours, this morning, during which time she suffered great oppression. On the renewal of the discharge she became again quite easy, and so remained during the day. Urinary discharge continues very scanty.

12*th.* Summoned hastily, and found her much alarmed, discharging blood mixed with water most profusely from the vagina; sometimes clots of blood alone. I immediately prescribed sacch. saturini and opium, with cold applications; enjoined the horizontal position, and absolute quietude. Upon examination I found the neck of the uterus as in the seventh month of pregnancy, and satisfied myself that there was neither entire nor partial implantation of the placenta over the mouth of the uterus; as well as that the discharge was from within the chorion, and not as I sometimes suspected from immense hydatids exterior to the membranes. The membranes being ruptured, I was able distinctly to feel one of the lower extremities of a fœtus.

13*th.* Discharge of blood continues, and although very copious, has but little effect in reducing vascular action. Stomach extremely irritatable; vomiting almost incessant; complains much of acidity. Gave alkalies freely.

14*th.* Vomiting more and more distressing; great præcordial uneasiness. No abatement, but rather an increase of the discharge from the uterus of blood and water. About five o'clock, P. M., called in great haste, and found, much to my relief, that labor had fairly commenced—it being the first time during the whole period of discharge, that the uterus showed any disposition to rid itself of its contents. Found the os tincæ dilating; nates of a fœtus presenting, which was delivered, weighing about three pounds. Uterine contractions soon returned, and a second fœtus was delivered, a vertex presentation, of about the same size as the first. Both lived several hours.

After labor commenced there was no discharge of either blood or water. The twins were contained in one common involucrum, and consequently there was but one placenta. Patient continued to vomit incessantly until the birth of the first child, when it ceased entirely, and she became comfortable immediately after delivery. About ten days after delivery, she voided a large quantity of purulent matter, which continued occasionally for two or three days.

In three weeks after her confinement the patient left this place for the South in good health and spirits.

*November* 18*th*, 1840.

